# The Anthocyanin Composition and Key Regulatory Genes Underlying the Color Variation Between Potato Tuber Bud Eyes and Periderm

**DOI:** 10.3390/plants15020226

**Published:** 2026-01-11

**Authors:** Xijuan Zhao, Chenxi Li, Shengxuan Liu, Zhuang Xiong, Xiaojian Zhang, Qian Li, Botao Song, Huiling Zhang, Xinxi Hu

**Affiliations:** 1 Key Laboratory for Vegetable Biology of Hunan Province, Yuelu Mountain Laboratory, Hunan Agricultural University, Changsha 410128, China; zhaoxijuan@hunau.edu.cn; 2 Urumqi Comprehensive Experimental Station, Academy of Agricultural Sciences of Xinjiang Uygur Autonomous Region, Urumqi 830013, China; 3 National Key Laboratory for Germplasm Innovation and Utilization of Horticultural Crops, Key Laboratory of Potato Biology and Biotechnology, Ministry of Agriculture and Rural Affairs, College of Horticulture and Forestry Sciences, Huazhong Agricultural University, Wuhan 430070, China; 2022305120061@webmail.hzau.edu.cn (C.L.); liu_shenxuan@webmail.hzau.edu.cn (S.L.); xxzz.hzau.edu.cn@webmail.hzau.edu.cn (Z.X.); liqianqian@mail.hzau.edu.cn (Q.L.); songbotao@mail.hzau.edu.cn (B.S.); 4 College of Horticulture and Plant Protection, Henan University of Science and Technology, Luoyang 471000, China; a18839321212@163.com

**Keywords:** potato, anthocyanin biosynthesis, *StbHLH14*, negative regulation

## Abstract

The pigmentation patterns of potato tubers are complex and diverse, often exhibiting significant tissue specificity. This study was conducted to elucidate the molecular mechanisms underlying the differential pigmentation in different parts of potato tubers using two cultivars, ‘Huashu 12’ and 15EM36-26, which exhibit opposite pigmentation patterns between the bud eyes and the tuber periderm. Metabolomic analysis revealed that cyanidin, pelargonidin, and malvidin are the key anthocyanin components responsible for the observed pigmentation differences. A total of 118 common differentially expressed genes in the differentially pigmented tissues of both cultivars were identified in transcriptomic analysis, including key structural genes of the anthocyanin biosynthesis pathway (such as *StPAL*, *StCHS*, and *StDFR*). Weighted gene co-expression network analysis was further employed to screen modules significantly correlated with pigmentation phenotypes, and 28 candidate genes associated with anthocyanin biosynthesis were identified. Expression validation demonstrated that the expression of *StbHLH14* was significantly higher in non-pigmented tissues compared to pigmented tissues. Functional analysis revealed that StbHLH14 can inhibit the activation of structural gene promoters (such as *StCHS* and *StDFR*) via the MYB transcription factor StAN2, thereby negatively regulating anthocyanin biosynthesis. This study unveils the metabolic and transcriptional basis of tissue-specific pigmentation in potato tubers and clarifies the negative regulatory role of *StbHLH14*.

## 1. Introduction

Anthocyanins, a class of water-soluble natural pigments widely present in plants, not only determine the vibrant colors of many flowers, fruits, and storage organs (such as tubers) [1], but have also garnered significant attention due to their strong antioxidant properties and potential health benefits for humans [2,3]. Moderate intake of anthocyanins can help reduce inflammation, inhibit bacteria, prevent cardiovascular and cerebrovascular diseases, and combat tumors [2,3]. The accumulation of anthocyanins in potato tubers, a globally important food crop, is closely related to their nutritional and economic value.

The biosynthesis of anthocyanins requires the involvement of a series of enzymes, such as phenylalanine ammonia-lyase (PAL), cinnamate-4-hydroxylase (C4H), 4-coumarate coenzyme A ligase (4CL), chalcone synthase (CHS), chalcone isomerase (CHI), flavanone 3-hydroxylase (F3H), flavonoid 3′-hydroxylase (F3′H), flavonoid 3′5′-hydroxylase (F3′5′H), dihydroflavonol 4-reductase (DFR), anthocyanidin synthase (ANS), and UDP-glucose:flavonoid-3-O-glycosyl transferase (UFGT). These enzymes are encoded by key genes involved in the anthocyanin biosynthesis pathway [4,5]. In potatoes, several key enzyme genes affecting anthocyanin biosynthesis have been successively cloned and functionally characterized. For instance, *StF3′5′H* controls the formation of purple pigments in tubers. Overexpression of *StF3′5′H* in the red-skinned cultivar ‘Desiree’ resulted in transgenic lines, with stems and tubers turning from red to purple [6]. Similarly, when the *StDFR* gene was introduced into ‘Prince Hairy’, a variety with white tubers and light blue flowers, the flower color of the transgenic lines changed to purple, while the tuber color remained white [7]. Furthermore, the expression level of the *StANS* gene is higher in colored genotypes than yellow ones. Overexpression of *StANS* in Desiree promoted anthocyanin biosynthesis in tubers [8].

The expression of structural genes is precisely regulated primarily by MYB, bHLH, and WD40 transcription factors, as well as protein complexes formed by these three components [1]. Among these, MYB transcription factors can directly interact with the promoters of structural genes to enhance or suppress their expression, thereby regulating anthocyanin biosynthesis. For instance, StAN2 activates the expression of *StF3H* and *StDFR* in potato by binding to the MYB-binding sites in their promoters, thereby promoting anthocyanin biosynthesis [9]. In grapes, VvMYBA specifically activates the expression of the downstream gene *UFGT*, regulating anthocyanin synthesis in red-skinned grapes [10].

The bHLH transcription factor family is the second-largest transcription factor family in plants [11]. These factors can directly regulate the expression of structural genes involved in anthocyanin biosynthesis, thereby influencing anthocyanin accumulation. For example, PavbHLH102 significantly promotes the accumulation of anthocyanins, particularly cyanidin-derived pigments, by directly activating key genes such as *PavF3H*, *PavF3H*, *PavDFR*, and *PavUFGT* [12]. Alternatively, bHLH proteins can indirectly regulate anthocyanin biosynthesis by interacting with MYB transcription factors, affecting their ability to bind target genes. Both B bHLH and R bHLH interact with the R2R3-MYB transcription factor C1 in maize to activate the promoter activity of the structural gene *DFR* [13]. Plant bHLH transcription factors can also negatively regulate anthocyanin biosynthesis through various molecular mechanisms. In maize, the bHLH protein IN1 inhibits anthocyanin accumulation by competitively binding to the MYB protein C1 against activators like R1/B1 or by forming inactive heterodimers [14]. In *Arabidopsis*, bHLH32 negatively regulates anthocyanin biosynthesis induced by low phosphate stress by binding to TTG1/GL3, thereby interfering with the function of the MBW complex [15]. Members of the bHLH subfamily IIId (BHLH3/13/14/17) additionally suppress jasmonate-induced anthocyanin accumulation by directly and competitively binding to the promoters of anthocyanin biosynthesis genes (such as DFR), thereby antagonizing the function of activating complexes like MYC2 or TT8/MYB75/PAP1 [16]. These studies demonstrate that bHLH repressors achieve precise negative regulation of anthocyanin biosynthesis in different species and signaling pathways by competitively binding, interfering with complex formation, or directly inhibiting the promoter activity of structural genes.

The pigmentation patterns of potato tubers are complex and diverse, often exhibiting significant tissue specificity. For example, the accumulation of different types and contents of anthocyanins in the periderm and bud eyes (with overlying leaf scars) leads to various tuber phenotypes, such as red, purple, and yellow. Although the anthocyanin biosynthesis pathway has been extensively studied in many plant species, the key metabolites, regulatory genes, and their specific mechanisms underlying the pigmentation differences in distinct parts (e.g., periderm vs. bud eyes) of potato tubers remain unclear. In particular, the precise regulatory networks governing different anthocyanin components and their underlying mechanisms have not been fully elucidated. Two potato genotypes, the cultivar Huashu 12 and the breeding line 15EM36-26, which exhibit opposite pigmentation patterns between their bud eyes and tuber periderm, were utilized in this study as genetic materials. The aim was to systematically uncover the formation mechanisms of tissue-specific pigmentation in potato tubers. We sought to identify the key anthocyanin components responsible for pigmentation differences, screen critical candidate genes regulating these differences, and elucidate the function and regulatory mechanisms of the key transcription factor *StbHLH14*. This was achieved by integrating targeted metabolomics, transcriptomics, and weighted gene co-expression network analysis (WGCNA). The findings are expected to provide new insights into the complex regulatory network of anthocyanin biosynthesis in potatoes and offer important theoretical foundations and genetic resources for the molecular breeding of potato varieties with high anthocyanin content.

## 2. Results

### 2.1. Analysis of Anthocyanin Composition in Different Tissues of Huashu 12 and 15EM36-26 Tubers

**Figure 1:**
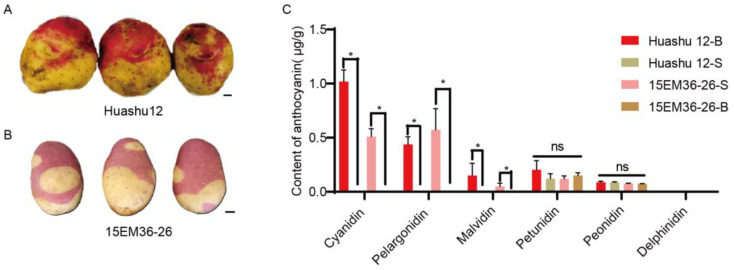
Potato tuber phenotypes and anthocyanin content. (**A**) Tubers of the cultivar Huashu 12. (**B**) Tubers of the strain 15EM36-26. Bar: 1 cm. (**C**) Anthocyanin content in the bud eyes and periderm of tubers from ‘Huashu 12’ and 15EM36-26. Huashu12-B and Huashu12-S indicate the pigmented bud eye region and the non-pigmented periderm region of Huashu 12, respectively; 15EM36-26-S and 15EM36-26-B indicate the pigmented periderm region and the non-pigmented bud eye region of 15EM36-26, respectively (two-tailed Student’s *t*-test; * indicates a significant difference at *p* < 0.05; ns indicates no significance; *p* > 0.05).

In this study, we observed that the tubers of Huashu 12 and 15EM36-26 exhibited opposite pigmentation patterns in the bud eyes and periderm tissues: the bud eyes of Huashu 12 tubers were red, while the periderm was yellow (Figure 1A), whereas in 15EM36-26, the bud eyes were yellow, and the periderm was red (Figure 1B). Targeted metabolomic analysis of anthocyanins was performed on different tuber tissues of both genotypes to further investigate the molecular basis underlying these differences. The results showed that delphinidin was not detected in any tissue of either genotype. While petunidin and peonidin were detected, their contents showed no significant differences, indicating that these three anthocyanins are not critical for the observed pigmentation variations. The key differences stemmed from the tissue-specific accumulation of cyanidin, pelargonidin, and malvidin. These anthocyanins were only detected in the pigmented tissues (bud eyes of Huashu 12 and periderm of 15EM36-26). Specifically, the highest cyanidin content was measured in the bud eyes of Huashu 12 (1.018 μg/g), followed by pelargonidin (0.438 μg/g). In contrast, neither pelargonidin nor cyanidin was detected in the non-pigmented tissues (periderm of Huashu 12 and bud eyes of 15EM36-26). Only a trace amount of malvidin (0.002 μg/g) was detected in the bud eyes of 15EM36-26, but its content was significantly lower than that in the pigmented tissues (Figure 1C). In summary, cyanidin, pelargonidin, and malvidin are the key anthocyanin components responsible for the pigmentation differences in the tubers of the two genotypes.

### 2.2. Analysis of Differentially Expressed Genes and Differential Metabolites

**Figure 2:**
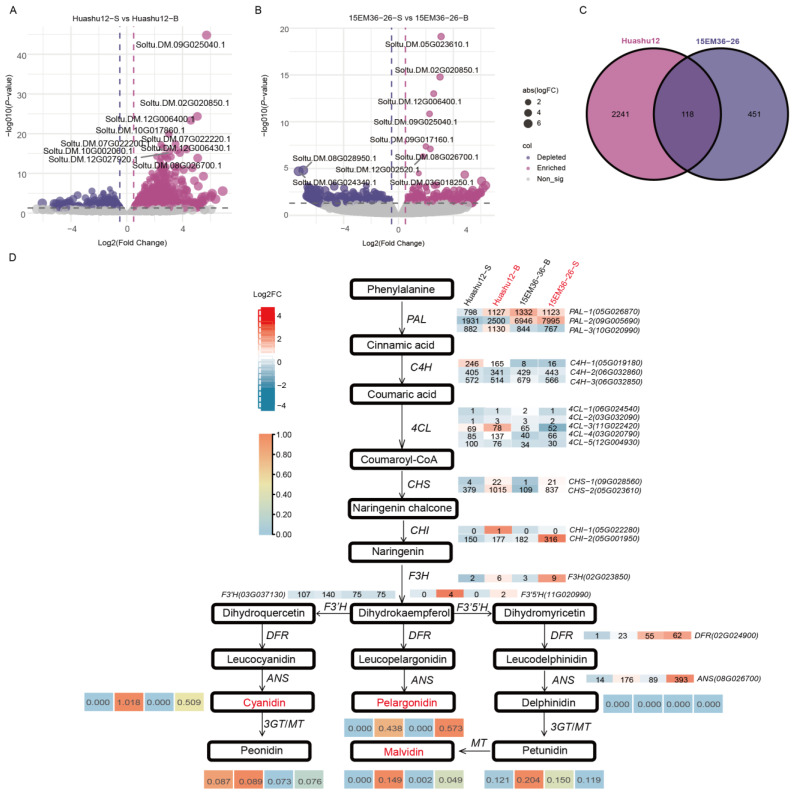
Analysis of differentially expressed genes and differential metabolites between ‘Huashu12’ and 15EM36-26. (**A**) Differentially expressed genes in ‘Huashu12’. (**B**) Differentially expressed genes in 15EM36-26. (**C**) Common differentially expressed genes shared by ‘Huashu12’ and 15EM36-26. (**D**) Analysis of differentially expressed genes and differential metabolites in the anthocyanin biosynthesis pathway. In the heatmap, gene expression levels are represented by standardized log10 (FPKM) values, while anthocyanin content is displayed as the actual measured values.

RNA-seq analysis of the bud eyes buds and periderm of tubers from ‘Huashu12’ and 15EM36-26 in this study to further elucidate the molecular regulatory mechanisms underlying anthocyanin biosynthesis in different tissues. The results revealed a total of 2359 differentially expressed genes (DEGs) between the bud eyes and periderm of ‘Huashu12’ (Figure 2A) and 569 DEGs in 15EM36-26 (Figure 2B). Venn diagram analysis showed that there were 118 common DEGs in the differentially pigmented tissues between the two genotypes (Figure 2C, Table S1). An anthocyanin biosynthesis pathway map was constructed based on KEGG pathway annotation and DEG information (Figure 2D) to explore the association between DEGs in the anthocyanin biosynthesis pathway and differential metabolites. In this pathway, significant differences were observed in the contents of cyanidin, pelargonidin, and malvidin between pigmented and non-pigmented tissues, accompanied by differential expression of several key structural genes, including *StPAL* (Soltu.DM.09G005690), *St4CL* (Soltu.DM.03G020790.1), *StCHS* (Soltu.DM.05G023610.1), *StCHI* (Soltu.DM.05G001950.1), *StF3H* (Soltu.DM.02G023850.1), *StF3′H* (Soltu.DM.03G037130.1), *StF3′5′H* (Soltu.DM.11G020990.1), *StDFR* (Soltu.DM.02G024900.1), and *StANS* (Soltu.DM.08G026700.1). Among these, the expression levels of *StPAL*, *StCHS*, *StDFR*, and *StANS* were significantly higher in pigmented tissues than in non-pigmented tissues. The expression pattern of *StC4H* (Soltu.DM.06G032860.1) was not entirely consistent between the two genotypes.

### 2.3. Weighted Gene Co-Expression Network Module Analysis

**Figure 3:**
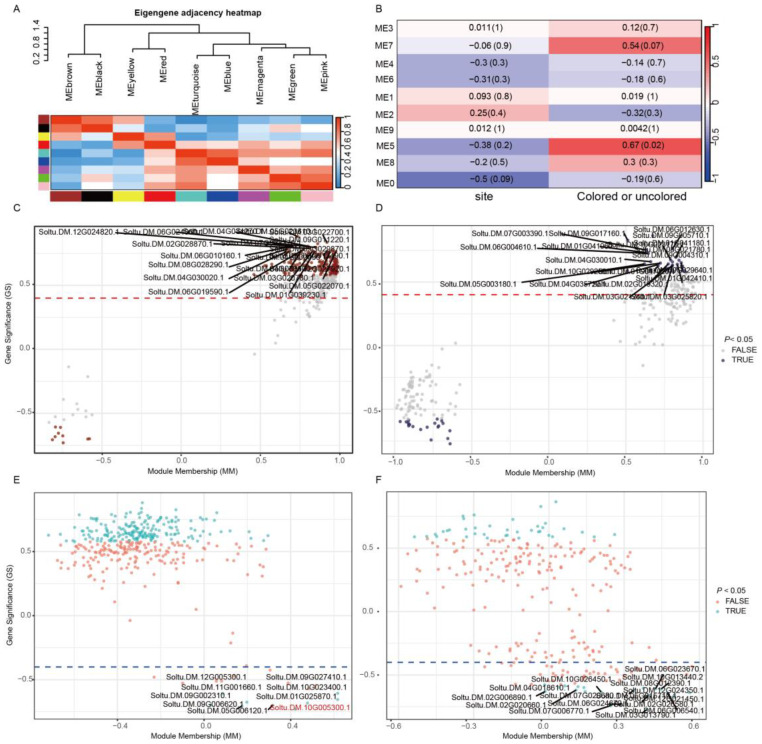
Weighted gene co-expression network analysis. (**A**) Module cluster analysis. (**B**) Correlation between modules and traits. The numbers in the boxes represent correlation coefficients, with the corresponding *p*-values indicated in parentheses. (**C**,**D**) Significant positively correlated genes in modules 5 and 7. (**E**,**F**) Significant negatively correlated genes in modules 5 and 7. The X-axis represents module membership (MM), and the Y-axis represents gene significance (GS) in (**C**–**F**).

WGCNA was performed based on the top 5000 genes ranked by expression level to further identify key genes regulating pigmentation differences in different tissues of potato tubers. The results showed that all genes were clustered into nine color modules (Figure 3A). Module–trait association analysis revealed that module 5 exhibited the highest correlation with the pigmentation or non-pigmentation phenotype (r = 0.67, *p* = 0.02), followed by module 7 (r = 0.54, *p* = 0.07). Based on the absolute values of module membership and gene significance, significantly positively and negatively correlated genes were further screened from modules 5 and 7, respectively. The results showed that module 5 contained 188 significantly positively correlated genes (Figure 3C, Table S2) and 9 significantly negatively correlated genes (Figure 3E, Table S2). In contrast, module 7 included 37 significantly positively correlated genes (Figure 3D, Table S2) and 20 significantly negatively correlated genes (Figure 3F, Table S2).

### 2.4. Expression Analysis of Genes Associated with Differential Pigmentation in Tuber Periderm

**Figure 4:**
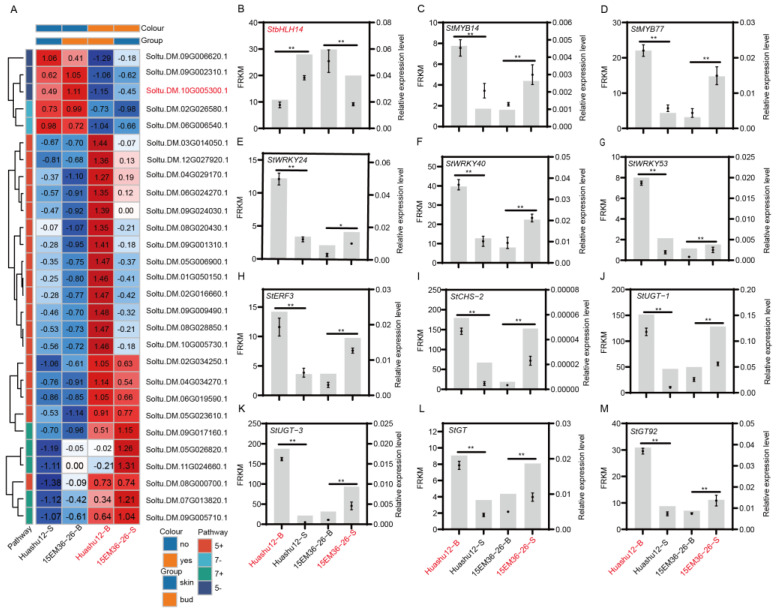
Comparative analysis of gene co-expression modules and RT-qPCR validation of module 5 and 7 significant genes. (**A**) Gene expression profiles of the significant module associated with differential coloration. (**B**–**M**) RT-qPCR validation of module 5 and 7 significant genes (two-tailed Student’s *t*-test; * indicates a significant difference at *p* < 0.05; ** indicates a significant difference at *p* < 0.01).

After functional annotation of the genes screened from modules 5 and 7, a total of 28 genes significantly associated with anthocyanin biosynthesis were identified. Further cluster analysis of these genes revealed that their expression patterns could be clearly divided into two categories (Figure 4A, Table S3). The first category includes genes with higher expression levels in non-pigmented tissues (Huashu12-S and 15EM36-26-B) than pigmented tissues (Huashu12-B and 15EM36-26-S). This category comprises five genes negatively correlated with the pigmentation phenotype, including three from module 5 (bHLH transcription factor Soltu.DM.10G005300.1, zinc finger Soltu.DM.09G006620.1, and RING-H2 Soltu.DM.09G002310.1) and two from module 7 (elongation factor Soltu.DM.02G026580.1 and Soltu.DM.06G006540.1). These genes may negatively regulate anthocyanin biosynthesis in potato tubers. The second category includes genes with higher expression levels in pigmented tissues than in non-pigmented tissues, comprising 23 genes positively correlated with the pigmentation phenotype. Among these, 18 are from module 5 and 5 from module 7. Representative genes include the anthocyanin biosynthesis structural gene Soltu.DM.09G017160.1, MYB transcription factor Soltu.DM.04G034270.1, ERF transcription factor Soltu.DM.06G019590.1, and WRKY transcription factor Soltu.DM.06G024270.1. These genes may function as positive regulators in anthocyanin synthesis and accumulation.

To further validate the expression patterns of these genes, 12 genes with relatively high expression levels were selected for quantitative analysis, including 7 transcription factors and 5 structural genes. The transcription factors were *StbHLH14* (Soltu.DM.10G005300.1), *StMYB14* (Soltu.DM.03G014050.1), *StMYB77* (Soltu.DM.04G034270.1), *StWRKY24* (Soltu.DM.09G009490.1), *StWRKY40* (Soltu.DM.06G024270.1), *StWRKY53* (Soltu.DM.08G028850.1), and *StERF3* (Soltu.DM.06G019590.1). The structural genes were *StUGT-1* (Soltu.DM.09G017160.1), *StUGT-3* (Soltu.DM.12G027920.1), *StCHS-2* (Soltu.DM.05G023610.1), *StGT* (Soltu.DM.08G000700.1), and *StGT92* (Soltu.DM.05G006900.1). The quantitative results showed that the expression of *StbHLH14* (Soltu.DM.10G005300.1) was significantly higher in non-pigmented tissues than in pigmented tissues, while the other 11 genes exhibited significantly higher expression levels in pigmented tissues than in non-pigmented tissues. The overall expression trends were consistent with the transcriptome data (Figure 4B–M).

### 2.5. StbHLH14 Negatively Regulates Anthocyanin Biosynthesis

**Figure 5:**
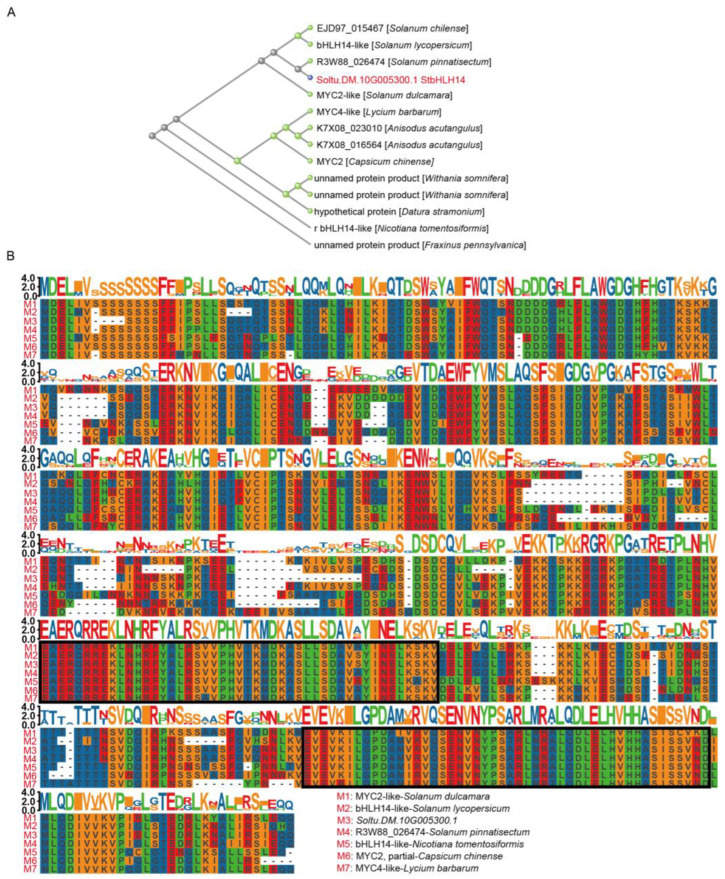
Phylogenetic analysis and multiple sequence alignment of StbHLH14. (**A**) Phylogenetic analysis of the key negatively correlated protein StbHLH14 from module 5. (**B**) Multiple-sequence alignment of StbHLH14.

To predict the regulatory role of *StbHLH14* in anthocyanin biosynthesis, phylogenetic clustering analysis was performed on StbHLH14 and its homologous proteins from 13 *Solanaceae* species, including tomato (*Solanum lycopersicum*) and bittersweet nightshade (*Solanum dulcamara*), as well as green ash (*Fraxinus pennsylvanica*). The results showed that all sequences could be divided into three main branches. The target protein StbHLH14 clustered closely with tomato bHLH14-like and bittersweet nightshade MYC2-like, with high node support values, indicating a close phylogenetic relationship (Figure 5A). Further multiple sequence alignment was conducted on StbHLH14, tomato bHLH14-like, bittersweet nightshade MYC2-like, and seven other homologous proteins. The results revealed high overall sequence consistency, particularly in the key functional domains, though notable divergence was observed in certain amino acid sequences and non-conserved regions (Figure 5B).

**Figure 6:**
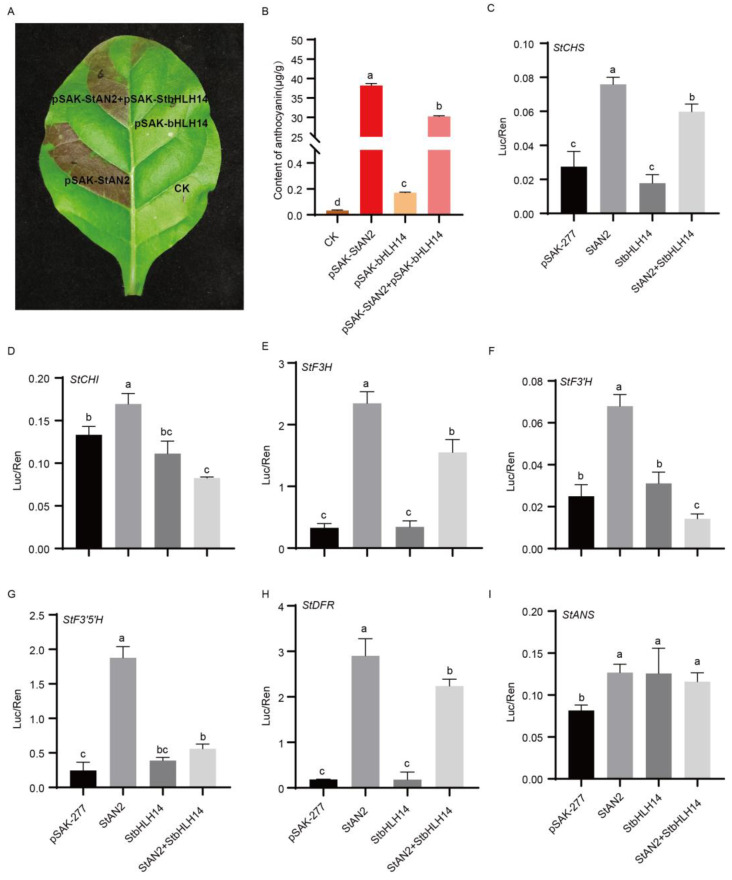
Regulation of StbHLH14 on structural genes. (**A**) Transient transformation injection of tobacco. (**B**) Anthocyanin content in tobacco leaves after injection. (**C**–**I**) Effect of StbHLH14 on the promoter activity of *StCHS*, *StCHI*, *StF3H*, *StF3′H*, *StF3′5′H*, *StDFR*, and *StANS*. The X-axis labels indicate different plasmid combinations: pSAK-277 (empty vector), StAN2, StbHLH14, and StAN2 + StbHLH14, which were co-transformed with the target promoter vector into tobacco. Different letters indicate significant difference between the different groups according to ANOVA (Tukey’s method, *p* < 0.05).

Based on existing studies, the bHLH factor JA-ASSOCIATED MYC2-LIKE1 (JAM1), which negatively regulates the jasmonic acid signaling pathway, can inhibit jasmonate-induced anthocyanin biosynthesis [17,18]. JAM1 (bHLH17) belongs to the Arabidopsis bHLH subfamily IIId, along with bHLH3, bHLH13, and bHLH14. Members of this subfamily not only antagonize the function of MYC2 on its target gene promoters but also inhibit the activation of the *DFR* promoter by the TT8/MYB75 complex [16]. Therefore, it is hypothesized that StbHLH14 performs functions similar to MYC2-like proteins and negatively regulates anthocyanin biosynthesis. To elucidate the role of the *StbHLH14* gene in anthocyanin biosynthesis, its CDS sequence was cloned from Huashu 12. The sequence is 1374 bp in length, encoding 457 amino acids. An overexpression vector was constructed and transiently expressed in the leaves of *Nicotiana tabacum*. The results showed that the tobacco leaves injected with StbHLH14 alone showed no significant difference compared with the control, while those injected with the anthocyanin positive regulator StAN2 alone turned distinctly purple. In contrast, the leaves still exhibited a clear purple phenotype when co-injected with both StAN2 and StbHLH14, but the intensity of pigmentation was lighter than that of leaves injected with StAN2 alone (Figure 6A). Anthocyanin content was consistent with these phenotypic observations (Figure 6B). When StAN2 was injected alone, anthocyanin content reached 38.18 μg/g, whereas co-injection of StAN2 and StbHLH14 significantly reduced anthocyanin accumulation. These results indicate that *StbHLH14* negatively regulates the StAN2-mediated anthocyanin biosynthesis pathway.

To further investigate the regulatory mechanism of *StbHLH14* in the anthocyanin biosynthesis pathway, a dual-luciferase reporter system was employed to examine the effect of StbHLH14 on the promoter activities of the structural genes of anthocyanin biosynthesis. The results showed that StAN2 could significantly activate the promoter activities of anthocyanin biosynthesis genes, including *StCHS*, *StCHI*, *StF3H*, *StF3′H*, *StF3′5′H*, *StDFR*, and *StANS*. StbHLH14 could enhance the promoter activities of *StANS*, while the other structural genes showed no significant difference compared with the control. However, when StAN2 and StbHLH14 were co-injected, the promoter activities of all these genes (except for *StANS*) were significantly reduced compared with injecting StAN2 alone (Figure 6C–I). These results demonstrate that StbHLH14 negatively regulates anthocyanin biosynthesis by inhibiting the activation of *StCHS*, *StCHI*, *StF3H*, *StF3′H*, *StF3′5′H*, and *StDFR* promoters by StAN2.

## 3. Discussion

The color of potato tubers is a crucial commercial trait primarily determined by the type and content of anthocyanins. The specific accumulation of anthocyanins in plant organs is a complex outcome of spatiotemporal gene expression regulation. The metabolic basis and molecular regulatory mechanisms underlying the color differences in distinct tuber regions (bud eyes and periderm) of two potato accessions (Huashu 12 and 15EM36-26) exhibiting opposite pigmentation patterns were systematically revealed in this study via the integration of metabolomics, transcriptomics, and molecular biology approaches.

### 3.1. Key Anthocyanin Composition and Tissue-Specific Accumulation Underlie Tuber Coloring Patterns

Potato tubers exhibit a rich spectrum of natural color phenotypes, ranging from white and yellow to red and deep purple. This variation in coloration across different tuber tissues is widespread among both cultivated varieties and wild species and is primarily attributed to significant differences in the types and quantities of anthocyanins accumulated in each tissue [19]. In this study, metabolomic analysis revealed that tissue-specific accumulation of cyanidin, pelargonidin, and malvidin is the direct cause of the color divergence between Huashu 12 (red bud eyes) and 15EM36-26 (red periderm). These anthocyanins were only detected in pigmented tissues and were nearly absent in non-pigmented areas (Figure 1C). This finding aligns with previous studies across various plant species, wherein specific anthocyanin glycosides, such as derivatives of cyanidin and pelargonidin, are recognized as key pigments responsible for red and pink coloration [20]. Furthermore, delphinidin was not detected in non-pigmented tissues of either accession, and no significant differences were observed in petunidin or peonidin content. This indicates that red pigmentation in potato tubers is predominantly governed by the cyanidin and pelargonidin pathways rather than the delphinidin synthesis pathway catalyzed by *StF3′5′H* [6]. The observed divergence in pigmentation patterns suggests a high degree of autonomy and specificity in anthocyanin biosynthesis regulation across different tuber tissues, which may be governed by distinct genetic or epigenetic programs.

### 3.2. The Structural Genes and Transcription Factors of the Anthocyanin Biosynthesis Pathway Are Regulated in a Coordinated Manner

Transcriptomic analysis was conducted to elucidate the molecular basis underlying the differential accumulation of key anthocyanins. KEGG pathway analysis revealed that multiple key structural genes (*StPAL*, *StCHS*, *StDFR*, *StANS*, and so on) in the anthocyanin biosynthesis pathway were significantly upregulated in pigmented tissues (Figure 2D). Previous studies have shown that *3GT2* and *CHS2* play specialized roles in the specific pigmentation of potato stamens, while *PAL*, *CHS1*, *DFR*, *ANS*, and *GST* are closely associated with anthocyanin accumulation in tubers [21]. These findings partially align with the results of this study. Collectively, these results indicate that anthocyanin biosynthesis depends on the coordinated activation of multiple steps within the pathway, rather than the independent action of a single gene [22].

The known major transcription factors involved in anthocyanin biosynthesis regulation include MYB, bHLH, WD40-repeat, WRKY, ERF, NAC, and other classes [23,24]. In this study, transcription factors from the MYB, bHLH, WRKY, and ERF families were also identified. Transcription factors can influence anthocyanin biosynthesis by regulating the expression of structural genes. For example, the MYB transcription factor StAN2 promotes anthocyanin accumulation in tubers and other tissues by activating the expression of *StDFR* and *StF3′5′H* [25], while StWRKY13 enhances anthocyanin biosynthesis in potato tubers by activating the expression of *StCHS*, *StF3H*, *StDFR*, and *StANS* [26]. Through qRT-PCR validation, it was found that *StMYB14*, *StMYB77*, *StWRKY24*, *StWRKY40*, *StWRKY53*, and *StERF3* were highly expressed in pigmented tissues, whereas *StbHLH14* showed higher expression in non-pigmented tissues (Figure 4B).

### 3.3. The Role and Mechanism of StbHLH14 as a Negative Regulator in Anthocyanin Biosynthesis

bHLH transcription factors primarily regulate anthocyanin biosynthesis through two distinct mechanisms. First, they directly modulate the promoter activity of structural genes to influence their expression. For instance, PavbHLH102 promotes anthocyanin accumulation by directly activating the expression of structural genes such as *PavF3H*, *PavF3H*, *PavDFR*, and *PavUFGT* [12]. Second, they regulate anthocyanin biosynthesis by forming complexes with MYB transcription factors. For example, both StbHLH1 and StJAF13 interact with StAN2 to promote anthocyanin biosynthesis in tobacco leaves [27]. *StbHLH14* was identified in this study as a bHLH transcription factor that negatively regulates anthocyanin biosynthesis (Figure 6A,B). StbHLH14 alone can specifically activate the promoter activity of the structural gene *StANS* while exhibiting no activating effect on the promoters of other structural genes (Figure 6C–I). This specificity may be attributed to the presence of core regulatory elements within the *StANS* promoter that can bind StbHLH14. When StbHLH14 is co-expressed with StAN2, the enhancement of *StANS* promoter activity shows no significant difference compared with their individual expression (Figure 6I). However, this co-expression significantly reduces the promotive effect of StAN2 on other structural genes (Figure 6C–H). This phenomenon may result from competition between StbHLH14 and positively regulating bHLH transcription factors, such as StAN1, for binding to StAN2, thereby reducing the formation of the MYB–bHLH–WD40 (MBW) complex that positively regulates anthocyanin biosynthesis. This indirectly suppresses the promoter activity of structural genes involved in anthocyanin biosynthesis, ultimately leading to decreased anthocyanin accumulation. This mechanism is similar to that of the homologous gene Arabidopsis bHLH17, which also inhibits the promoter activity of *DFR* by forming a complex with TT8/MYB75 [17]. However, the precise regulatory mechanism requires further experimental validation. This discovery provides new insights into the fine-tuned regulation of anthocyanin biosynthesis.

StbHLH14 exhibits lower expression levels in non-pigmented tissues and higher expression levels in pigmented tissues (Figure 1 and Figure 4B). Functional analyses further demonstrate that StbHLH14 acts as a negative regulator of anthocyanin biosynthesis. This expression pattern suggests that the spatially specific inhibitory effect of StbHLH14 contributes to the differential accumulation of anthocyanin between tissues. The tissue-specific expression pattern of StbHLH14 may be caused by differential regulation upstream of the gene. Tissue-specific DNA methylation or histone modifications could modulate chromatin accessibility at the StbHLH14 locus, which may lead to differential transcriptional activity. The expression of StbHLH14 may be integrated into broader developmental or hormone-signaling networks (such as light, jasmonate, or abscisic acid pathways) that differ between tissues, leading to spatially distinct transcriptional outputs. Of course, this speculation needs further experimental verification.

## 4. Materials and Methods

### 4.1. Plant Materials

The tubers of Huashu 12 exhibit red bud eyes and yellow periderm, while the tubers of 15EM36-26 (Huashu 12 × Huashu 1) display yellow bud eyes and red periderm. The mature tubers of Huashu 12 and 15EM36-26 were harvested, the tuber surfaces were thoroughly cleaned, and separate samples were collected from the bud eyes and periderm tissues of each genotype. Three biological replicates were prepared for each genotype, with each replicate consisting of five healthy tubers of uniform size, and samples from these five tubers were pooled as one mixed sample. The samples were immediately flash-frozen in liquid nitrogen and stored at −80 °C. The same set of samples was utilized for anthocyanin content determination, RNA sequencing, and quantitative analysis.

For transient expression analysis, *Nicotiana tabacum* and *Nicotiana benthamiana* plants for dual-luciferase assays were grown under greenhouse conditions until the 5–6 leaf stage, at which point they were ready for agroinfiltration.

### 4.2. Anthocyanin Extraction and Quantitative Analysis

The extraction of anthocyanin mixtures was performed according to the Chinese Agricultural Industry Standard (NY/T 2640-2014) [28]. The specific procedure was as follows: 0.1 g of powdered sample was weighed and mixed with 1.0 mL of methanol solution containing 1% formic acid, followed by overnight extraction at 4 °C in the dark. The extract was centrifuged at 10,000× *g* for 10 min and then filtered through a 0.22 μm microporous membrane. Quantitative analysis of anthocyanins was conducted using high-performance liquid chromatography (HPLC) based on previously described methods [29]. The anthocyanin standards used included peonidin, delphinidin, malvidin, petunidin, pelargonidin, and cyanidin, all prepared in methanol as the solvent.

### 4.3. RNA Extraction and Transcriptome Analysis

Total RNA extraction was performed following the method described in the previous literature [26]. The integrity of RNA was assessed using the Fragment Analyzer 5400 system (Agilent Technologies Inc., Santa Clara, CA, USA). The Illumina^®^ sequencing libraries were prepared using the NEBNext^®^ Ultra™ RNA Library Prep Kit (New England Biolabs Inc., Ipswich, MA, USA) according to the manufacturer’s instructions, with index codes added to distinguish sequences from different samples. mRNA was isolated from total RNA by employing poly-T oligo-conjugated magnetic beads. Fragmentation was conducted with divalent cations at elevated temperature in NEBNext First Strand Synthesis Reaction Buffer (5×). First-strand cDNA synthesis was performed using random hexamer primers and M-MuLV Reverse Transcriptase, followed by second-strand synthesis with DNA Polymerase I and RNase H. Remaining overhangs were blunt-ended through exonuclease/polymerase activities. Following adenylation of the 3 ends of DNA fragments, NEBNext Adaptors featuring a hairpin loop structure were ligated to facilitate hybridization. PCR amplification was then carried out with Phusion High-Fidelity DNA polymerase, universal PCR primers, and an index (X) primer. The resulting PCR products were purified (AMPure XP system), and library quality was evaluated on an Agilent Bioanalyzer 2100 system (Agilent Technologies Inc., Santa Clara, CA, USA). Finally, the prepared libraries were sequenced on an Illumina HiSeqTM 2000 platform (Illumina, San Diego, CA, USA).

### 4.4. Sequence Data Filtering, De Novo Assembly, and Annotation

The raw files obtained from the Illumina platform were converted into initial data through base calling. Quality control processing was performed on the raw reads: adapter sequences were removed, reads containing more than 5% poly-N were filtered out, and low-quality sequences were discarded. After processing, the raw sequences were transformed into high-quality clean reads. Subsequently, the clean reads were aligned to the potato DM reference genome transcriptome (version 6.1, http://spuddb.uga.edu/dm_v6_1_download.shtml, accessed on 16 April 2024) using the Salmon software (version 1.3.0). Gene function annotation was based on the NR (Non-Redundant) Protein Sequence Database, SwissProt/UniProt Plant Proteins, COG/KOG (Cluster of Orthologous Groups of proteins), Gene Ontology (GO), and KEGG.

### 4.5. Differential Expression Analysis

The raw gene expression counts were first normalized. Subsequently, differential expression analysis was performed using DESeq2, a statistical model based on the negative binomial distribution [30], to calculate log2 fold changes and *p*-values. The final criteria for screening differentially expressed genes were set as absolute log2 fold change > 1 and false discovery rate (FDR) < 0.05. KEGG pathway enrichment analysis of differentially expressed genes (DEGs) was performed using KOBAS software (version 3.0).

### 4.6. Weighted Gene Co-Expression Network Analysis

Based on the gene expression data from all samples, weighted correlation coefficients were used to construct relationships between genes, and a scale-free topological network was established accordingly. The dynamic tree-cutting method was employed to identify gene modules with high co-expression characteristics. The correlation between each module’s eigengene and the target phenotypic data was calculated to screen for key modules significantly associated with specific traits. Subsequently, functional enrichment analysis was performed on the genes within these modules [31]. The moduleEigengenes function was used to calculate module eigengenes, and the plotEigengeneNetworks function was applied to generate heatmaps visualizing the network of module eigengenes.

### 4.7. Real-Time Fluorescence Quantitative PCR (RT-qPCR)

Primers for RT-qPCR were designed using the TBtools software (version 2.363), and their sequences are listed in Table 1. RT-qPCR analysis was performed using the CFX96™ Real-Time PCR System (Bio-Rad, Hercules, CA, USA) and the TransStart Top Green qPCR SuperMix Kit (TransGen Biotech, Beijing, China). The potato *EF1α* gene (GenBank accession number: AB061263) was used as the reference gene, and the relative expression levels of the target genes were calculated using the multi-reference gene normalization method described by Vandesompele [32].

### 4.8. Phylogenetic Evolution and Amino Acid Sequence Alignment

Target proteins and their homologous sequences were downloaded from the NCBI database. Multiple sequence alignment was performed using the MUSCLE (v3.8) program [33]. The alignment results were manually inspected and trimmed to remove poorly aligned regions [34]. The optimal amino acid substitution model was determined using tools such as ProtTest (version 3.4.2) [35]. Based on this model, a phylogenetic tree was constructed using the maximum likelihood method implemented in PhyML software (version 3.1) [36]. The reliability of the tree topology was assessed through bootstrap analysis with 1000 replicates [37]. Visualization of the phylogenetic tree was performed using tools such as FigTree (v1.4.4).

### 4.9. Vector Construction and Transient Expression Analysis

Based on the sequences from the potato genome database, the open reading frames (ORFs) of the *StAN2* and *StbHLH14* genes were amplified from the tuber periderm cDNA of Huashu 12. The ORFs of *StAN2* and *StbHLH14* were separately cloned into the pSAK-277 vector using *Eco*RI restriction sites. The resulting recombinant plasmids, pSAK-StAN2 and pSAK-StbHLH14, were submitted to Sangon Biotech (Shanghai) Co., Ltd. (Shanghai, China) for sequencing verification. Transient expression of pSAK-StAN2 and pSAK-StbHLH14 in *Nicotiana tabacum* was analyzed following the method described in the previous literature [38]. The sequences of the primers pSAK-StAN2-F/R and pSAK-StbHLH14-F/R are detailed in Table 1.

The promoter sequences of *StCHS*, *StCHI*, *StF3H*, *StF3′H*, *StF3′5′H*, *StDFR*, and *StANS* were isolated from Huashu 12. The sequences of the primers used are detailed in Table 1. These promoters were cloned into the pGreenII0800-LUC vector using *Kpn*I and *Nco*I restriction sites. Dual-luciferase assays were performed in *Nicotiana benthamiana* following established methods [26,27], with the pGreenII0800-LUC empty vector serving as the negative control. The plasmids pSAK-StAN2 and pSAK-StbHLH14 were co-infiltrated in pairs with pGreenII0800-StCHS, pGreenII0800-StCHI, pGreenII0800-StF3H, pGreenII0800-StF3′H, pGreenII0800-StF3′5′H, pGreenII0800-StDFR, and pGreenII0800-StANS. After infiltration, the tobacco plants were incubated in darkness for 12 h and then transferred to a growth chamber under a 16 h light/8 h dark photoperiod. Samples were collected 3 days after infiltration, flash-frozen in liquid nitrogen, and stored at −70 °C. Finally, firefly luciferase (LUC) and renilla luciferase (REN) activities were measured using the Dual-Luciferase^®^ Reporter Assay System (Promega Corporation, Madison, WI, USA).

### 4.10. Statistical Analysis

Anthocyanin content analysis, RT-qPCR analysis, and transient transformation promoter activation analysis data are expressed as the mean (±SE) of three biological replicates. All data in this study were analyzed using two-sided Student’s *t*-test or one-way analysis of variance (ANOVA) to determine the statistical significance with GraphPad Prism v.9.0.

## 5. Conclusions

The tissue-specific anthocyanin accumulation mechanism in potato tubers was systematically elucidated in this study through the integration of multi-omics and molecular biology experiments. Targeted metabolomics revealed that the tissue-specific accumulation of cyanidin, pelargonidin, and malvidin in pigmented regions is the direct cause of the phenotypic differences. A total of 28 key genes were identified in differential expression and WGCNA analyses, among which 23 were highly expressed in pigmented tissues and played positive regulatory roles, while 5 were highly expressed in non-pigmented tissues and were potential negative regulators. Twelve of these genes were validated with qRT-PCR. Functional studies revealed that StbHLH14 finely tunes anthocyanin biosynthesis by inhibiting StAN2-mediated activation of downstream structural gene promoters. This confirms the core negative regulatory role of *StbHLH14* within this network. These findings provide new insights into the mechanisms underlying pigmentation in plant organs and establish an important theoretical foundation for improving color quality and breeding high-anthocyanin varieties in potatoes and other crops.

## Figures and Tables

**Table 1:** The primer sequences for RT-qPCR and the constructing vectors.

Primer Name	Gene ID	Forward (5′–3′)	Reverse (5′–3′)
EF1α	AB061233	ATTGGAAACGGATATGCTCCA	TCCTTACCTGAACGCCTGTCA
StUGT-1	Soltu.DM.09G017160.1	GGAAGAAACAGGGCTGCCT	TGTTGCTGCACCCAACCT
StUGT-3	Soltu.DM.12G027920.1	GGGTCGACTTAGCCCGTG	CCCCGTTTCTTCCCACCC
StCHS-2	Soltu.DM.05G023610.1	TTTGGTGATGGGGCAGCC	AGCCCAACTTCGCGAAGG
StGT	Soltu.DM.08G000700.1	TTACGGGCCTCTTCCCCA	CCTCGACAATCCTCGGGC
StGT92	Soltu.DM.05G006900.1	CGTCACCAGGAATTGCGG	TGTGTCTCTCGGGGCTCT
StbHLH14	Soltu.DM.10G005300.1	GGCAACACGCGAAACACC	GGAACAACAGAACGCAGAGC
StMYB14	Soltu.DM.03G014050.1	AAGGGGGCATGGTCTCCA	CCGTTCTTGAAAGCCCTGC
StMYB77	Soltu.DM.04G034270.1	GCGTTACGGAGCTCGGAA	TCCGCCGGAGAAAATGGC
StWRKY24	Soltu.DM.09G009490.1	TGCAGCACGTGGTAGTGG	GGCCTTGGAACTACTGGCA
StWRKY40	Soltu.DM.06G024270.1	CACCTGGTTGCCCTGTCA	ATCGGCTGGCAGTGGAAG
StWRKY53	Soltu.DM.08G028850.1	GGGCAACAAAGCAAGTGCA	TGTAGCAGCTGCAGCACA
StERF3	Soltu.DM.06G019590.1	CCGGACCCCCTACATTGC	TCAGTCCCAAACAAGTCGGG
pSAK-StAN2	Soltu.DM.10G020850.1	TAGTGGATCCAAAGAATTCATGAGTACTCCTATGATGTGTA	CGAGAAGCTTTTTGAATTCCTAATTAAGTAGATTCCATATATC
pSAK-StbHLH14	Soltu.DM.03G014050.1	TAGTGGATCCAAAGAATTCAGCTTCCACCTTCATCTTTCTCC	CGAGAAGCTTTTTGAATTCTTACTGCTGCTCAATGCTTCT
Prom-StCHS	Soltu.DM.05G023610.1	ATAGGGCGAATTGGGTACCCCTACTTAACAATCAAACACAACAA	TTTTTGGCGTCTTCCATGGCGTGTTTTTTTTTTTACTAAGATTT
Prom-StCHI	Soltu.DM.05G001950.1	ATAGGGCGAATTGGGTACCTCACTAACCTGAGAAGTAGGACGAG	TTTTTGGCGTCTTCCATGGATCAGTTGTATTATTACCAGAAGAGGAG
Prom-StF3H	Soltu.DM.02G023850.1	ATAGGGCGAATTGGGTACCTTGACATGTTTTTTTTTTAGCTAGG	TTTTTGGCGTCTTCCATGGGTTCAAAAGAGTTATGAGGTGCC
Prom-StF3′H	Soltu.DM.03G037130.1	ATAGGGCGAATTGGGTACCAAAAAAGGCTAGTCAAATGGGATAA	TTTTTGGCGTCTTCCATGGCGGGCCATTGATGCAGTG
Prom-StF3′5′H	Soltu.DM.11G020990.1	ATAGGGCGAATTGGGTACCATGTAAAAAATACGATAACAAAAAGT	TTTTTGGCGTCTTCCATGGCAACATGTGGCATTGAACCT
Prom-StANS	Soltu.DM.08G026700.1	ATAGGGCGAATTGGGTACCTATTGTGACTTTAGCTTTCATGATC	TTTTTGGCGTCTTCCATGGTGTTACGCGGAGTACTTATTTAGA
Prom-StDFR	Soltu.DM.02G024900.2	ATAGGGCGAATTGGGTACCCGCTATGTTATTGTTAAGGGTG	TTTTTGGCGTCTTCCATGGCAGAAATGAGAGGAAAAAAGAGTC

## Data Availability

The original contributions presented in this study are included in the article/supplementary material. Further inquiries can be directed to the corresponding authors.

## Supplementary Materials

The following supporting information can be downloaded from https://www.mdpi.com/article/10.3390/plants15020226/s1. Table S1: Genes used in Venn diagram analysis; Table S2: Genes of modules 5 and 7 in Figure 3; Table S3: The FPKM of 28 module genes in Figure 4.
